# Supplementary material for: Neoadjuvant PD-1 blockade induces T cell and cDC1 activation but fails to overcome the immunosuppressive tumor associated macrophages in recurrent glioblastoma
Source: Nat Commun. 2021 Nov 26;12:6938. doi: 10.1038/s41467-021-26940-2 (PMC8626557; doi:10.1038/s41467-021-26940-2)
Supplement: Supplementary file 2 — Supplementary Information [file 41467_2021_26940_MOESM2_ESM.pdf]

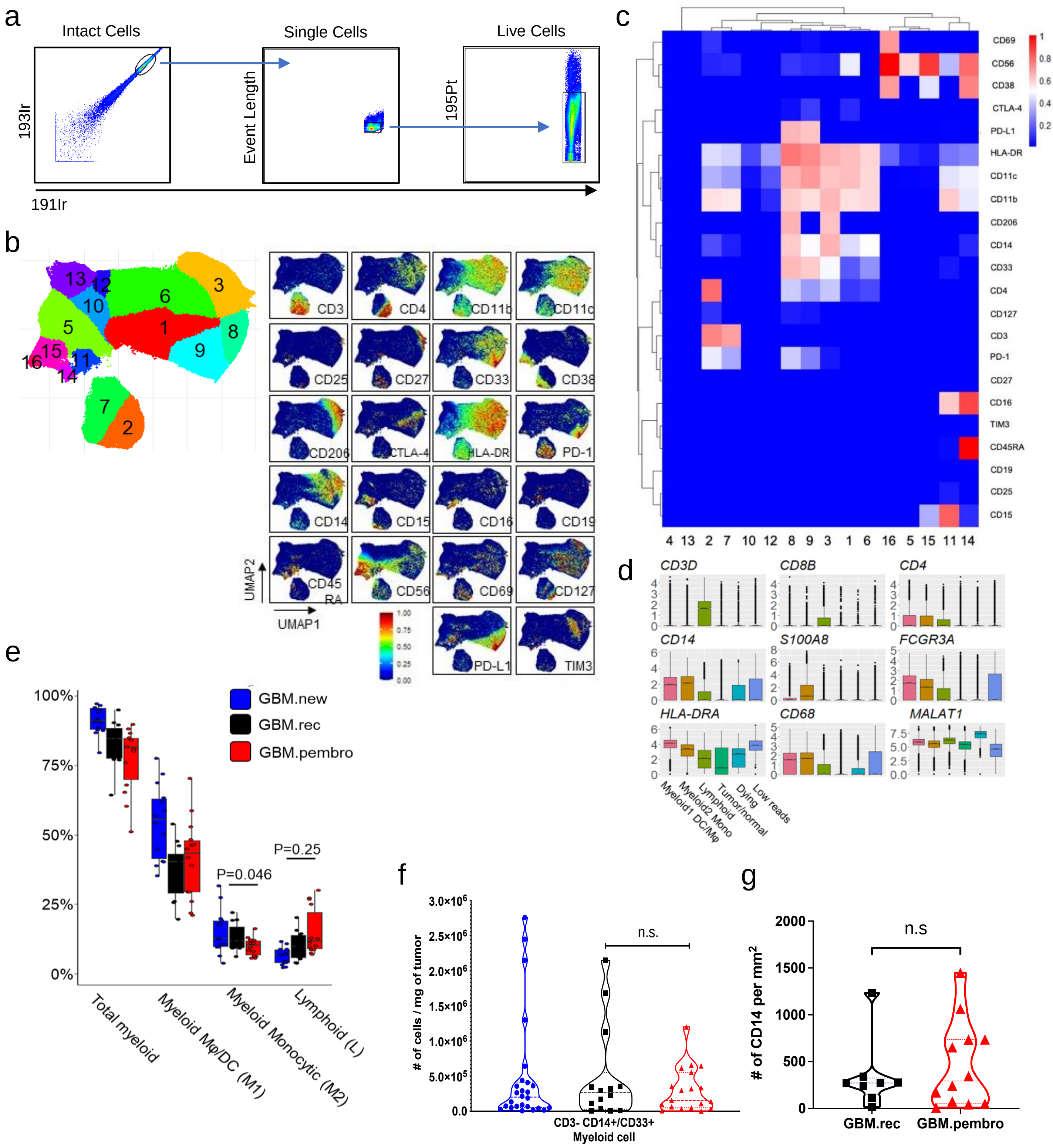

Supplementary Figure 1. Clustering of GBM-derived CD45+ cells based on the CyTOF and scRNAseq profiles.

a) Representative gating of raw fcs files to analyze the live intact single cells for subsequent CyTOF analysis.

b) (top) The clustering of tumor-infiltrating CD45+ cells analyzed on the 22 common marker CyTOF panel projected onto the UMAP space (n=1,067,057 cells from 63 total patients: 26 GBM.new, 17 GBM.rec, 20 GBM.pembro). The top large UMAP island is occupied by the different myeloid clusters while the bottom smaller island comprises the different lymphoid clusters. (bottom) UMAP plots of marker intensities on the TILs on the 22 marker CyTOF antibody panel

c) Heatmap of marker intensities on the CD45's on the 22 marker CyTOF antibody panel.

d) Expression of marker genes of distinct cell clusters derived from the scRNAseq data of GBM samples (n=156,766 cells from 40 total patients: 14 GBM.new, 12 GBM.rec, and 14 GBM.pembro).

e) The proportion of the cells in each cluster across the three groups of GBM samples (blue: GBM.new, black: GBM.rec, red: GBM.pembro).

f) The number of CD3- CD14/CD33 myeloid cells per mg of tumor across different tumor groups. Colors are the same as (e).

g) The number of CD14+ cells per mm^2 of tissue section for GBM.rec (black squares) and GBM.pembro (red triangles) samples. Images were collected and analyzed for 8 GBM.rec and 12 GBM.pembro patients.

P values were calculated using a two-sided Wilcoxon rank sum test. Source data are provided as a Source Data file. In all boxplots, the median is indicated by the line within the box and the 25th and 75th percentiles indicated by the lower and upper bounds of the box. The upper and lower lines above and below the boxes represent the whiskers.

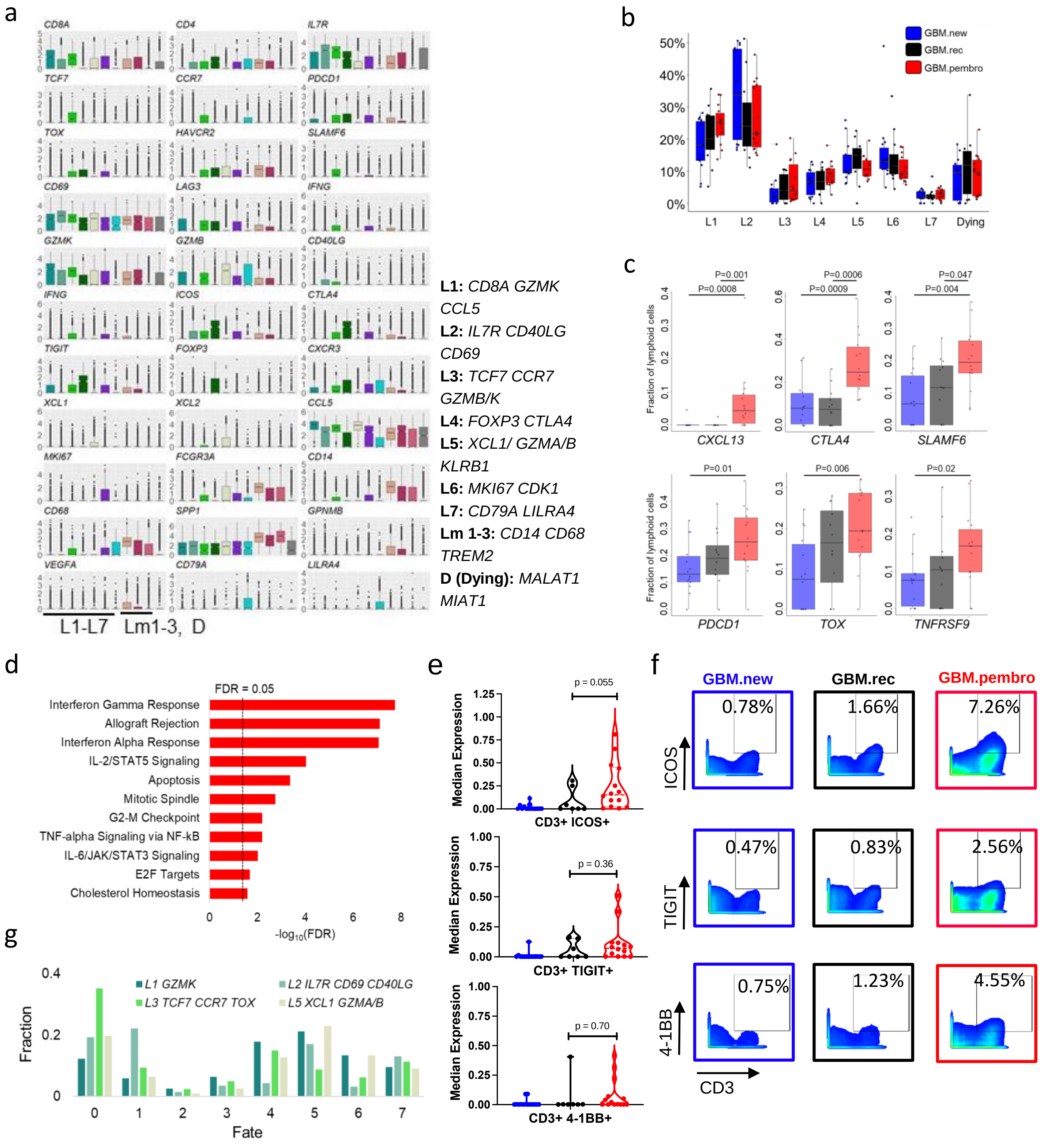

Supplementary Figure 2 – Identification of different lymphoid subpopulations from scRNAseq.
a) Expression of various genes across the different clusters used to identify the lymphoid populations in the TILs (n=14,322 cells from 40 total patients: 14 GBM.new, 12 GBM.rec, and 14 GBM.pembro).
b) The sample level proportions of each lymphoid cluster in (a) across GBM.new (blue), GBM.rec (black) and GBM.pembro (red).
c) The fraction of lymphoid cell expressing indicated transcripts in GBM.new, GBM.rec and GBM.pembro samples.
d) MSigDB Hallmark genesets showing significant overlap with the union of the genes upregulated in the GBM.pembro group across all lymphoid clusters (FDR values, two-sided fisher exact test).
e) Change in median expression of CD278 (ICOS), TIGIT, and 4-1BB (CD137) across all CD3+ clusters. Colors and statistical analysis are the same as (b) and (c).
f) Marker expression CD278 (ICOS), TIGIT, and 4-1BB (TNFRSF9) in the combined CyTOF data of the CD3+ T cells.
g) The fractions of CD8 T cells from the different Seurat clusters in (a) occupying the different T cell fates defined by the Monocle 2's pseudotime trajectory.
P values were calculated using a two-sided Wilcoxon rank sum test. Source data are provided as a Source Data file. In all boxplots, the median is indicated by the line within the box and the 25th and 75th percentiles indicated by the lower and upper bounds of the box. The upper and lower lines above and below the boxes represent the whiskers. The gene set enrichment P values were calculated using two-sided Fisher exact test with FDR adjustment for multiple comparisons.

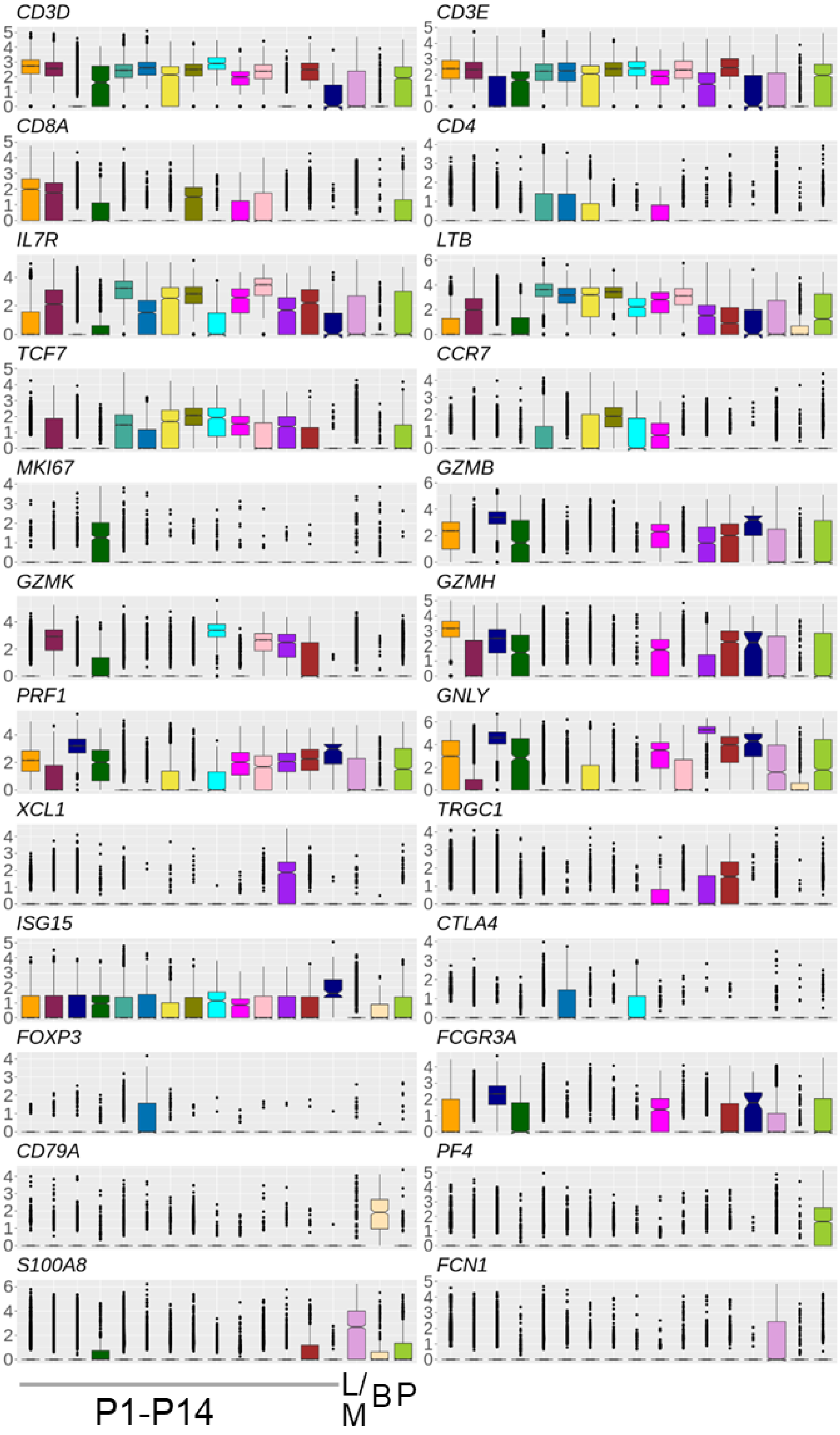

Supplementary Figure 3 – Expression of lymphoid markers in the PBMC scRNAseq
Expression of various genes across the different clusters that were used to identify the T cell populations in the PBMC (n=56,444 cells from 5 GBM.rec and 8 GBM.pembro patients and from 2 healthy donor controls). In all boxplots, the median is indicated by the line within the box and the 25th and 75th percentiles indicated by the lower and upper bounds of the box. The upper and lower lines above and below the boxes represent the whiskers.

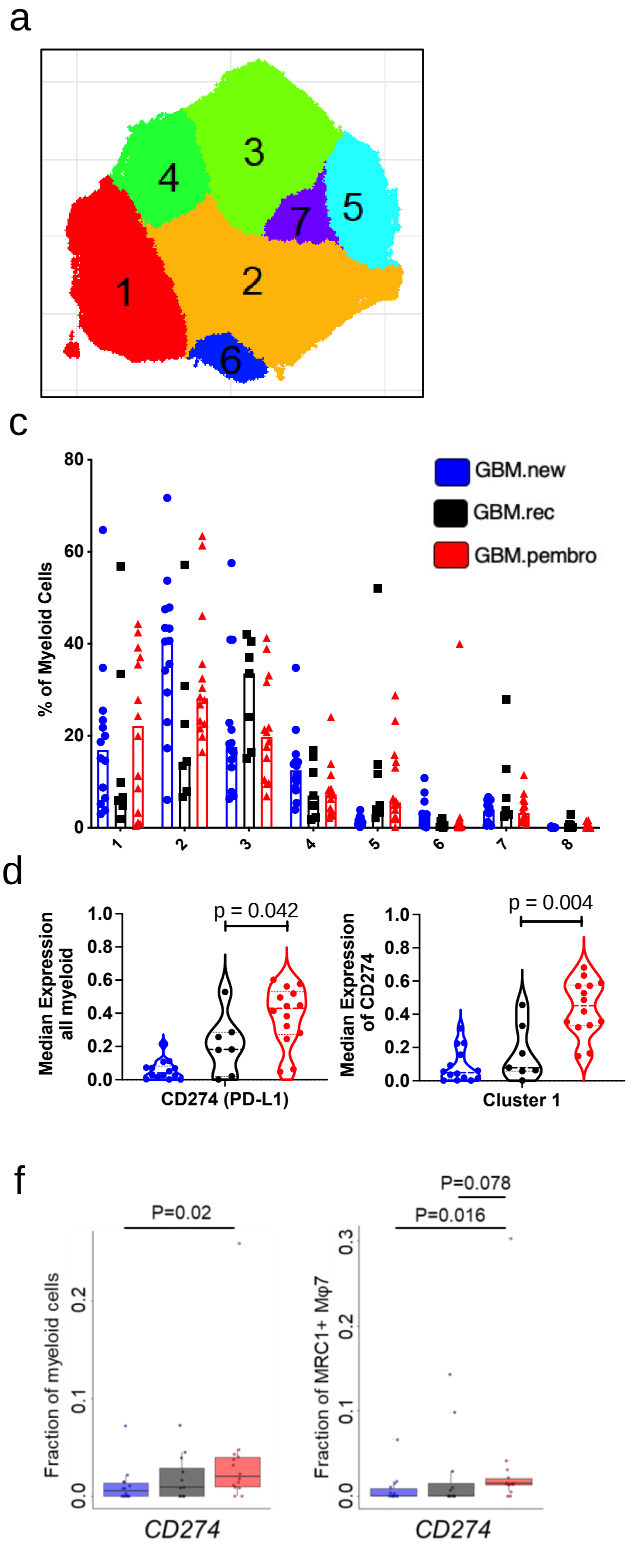

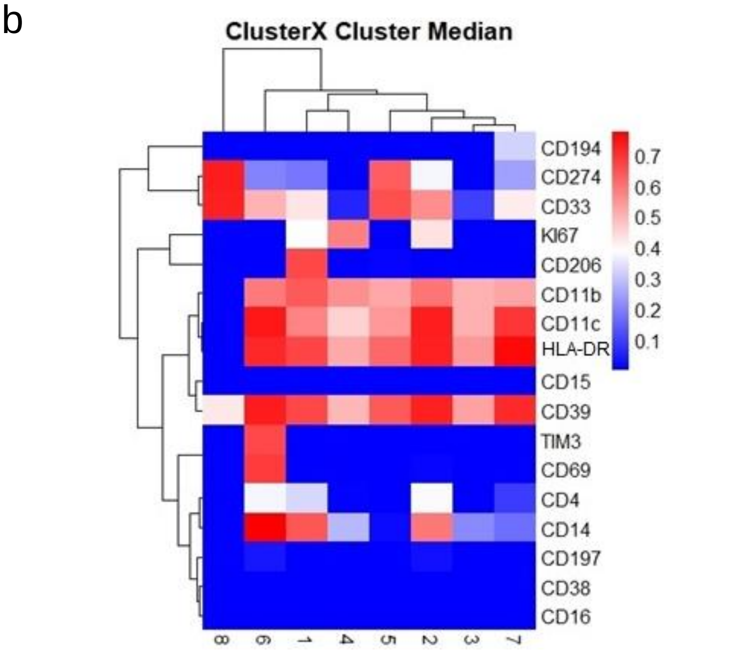

Cluster Identity	
c8	CD33+ PD-L1+ HLA-DR- Lin- (early myeloid progenitor)
c6	CD33+ CD14+ CD39+ CD11b/c+ HLA-DR+ (mono/Mφ/DC)
c1	CD33+ CD14+ CD206+ CD39+ HLA-DR+ Ki67^{mid} (Mature Mφ)
c4	CD14^{mid} CD39^{mid} Ki67+ HLA-DR^{mid} (monocyte/Mφ, proliferating)
c5	CD33+ CD11b+ CD11c+ PD-L1+ HLA-DR+ (early myeloid)
c2	CD33^{mid} CD14+ CD39+ PD-L1^{mid} CD11b/c+ HLA-DR+ (mono/Mφ/DC)
c3	CD14^{mid} CD39^{mid} HLA-DR^{mid} (monocyte/Mφ)
c7	CD33^{mid} CD14^{mid} CD39+ CD11c+ HLA-DR+ (early monocyte/DC)

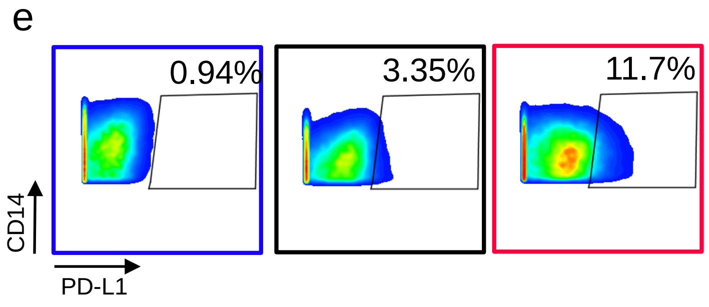

Supplementary Figure 4 – Myeloid cell clustering using the 34 marker CyTOF panel and scRNAseq show upregulation of PD-L1 with neo-aPD1.

a) UMAP projections showing specific marker intensities of the markers in the 34 marker CyTOF antibody panel (Supp. Data 1).

b) Heatmap of the marker expression intensities in each cluster. The combination of markers used to identify each cluster are listed in the table below the heatmap.

c) The percentage of tumor infiltrating myeloid cells within each cluster from (a) across different tumor conditions (blue circles: GBM.new, black squares: GBM.rec, red triangles: GBM.pembro).

d) Median CD274 (PD-L1) expression across all clusters (left) and in cluster 1 (right) separated by tumor diagnoses. Colors the same as (c).

e) Marker expression for CD14 and CD274 (PD-L1) in the combined, batch-corrected CYTOF data. Colors are the same as (c). Percentages represent the percent of CD14+ cells.

Supp. Dataf) Median CD274 (PD-L1) mRNA expression in all myeloid cells (left) or only in Mϕ7 cluster (right). Colors the same as (c) (n=72,492 cells from 40 total patients: 14 GBM.new, 12 GBM.rec, and 14 GBM.pembro). P values were calculated using a two-sided Wilcoxon rank sum test. Source data are provided as a Source Data file or Supp. Data 7. In all boxplots, the median is indicated by the line within the box and the 25th and 75th percentiles indicated by the lower and upper bounds of the box. The upper and lower lines above and below the boxes represent the whiskers.

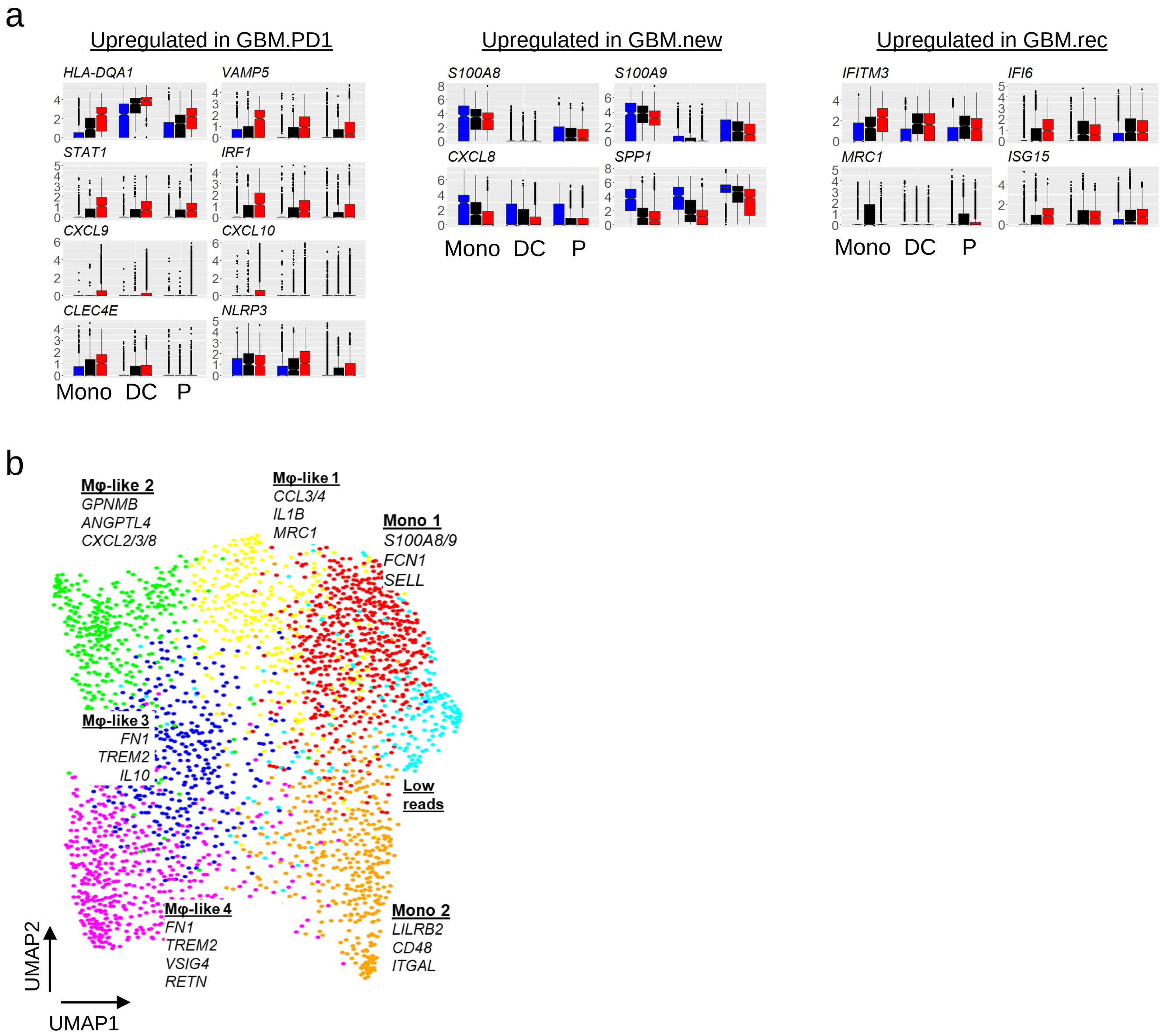

Supplementary Figure 5 – Increased activity of IL10+ immunosuppressive monocytic populations

a) Differentially expressed genes across the different conditions in the monocyte, DC, and proliferating myeloid clusters (blue: GBM.new, black: GBM.rec, red: GBM.pembro; n=2,677 cells from from 40 total patients: 14 GBM.new, 12 GBM.rec, and 14 GBM.pembro).

b) A UMAP projection of the monocytic compartment of the 40 patient samples that were analyzed using scRNAseq.

In all boxplots, the median is indicated by the line within the box and the 25th and 75th percentiles indicated by the lower and upper bounds of the box. The upper and lower lines above and below the boxes represent the whiskers.

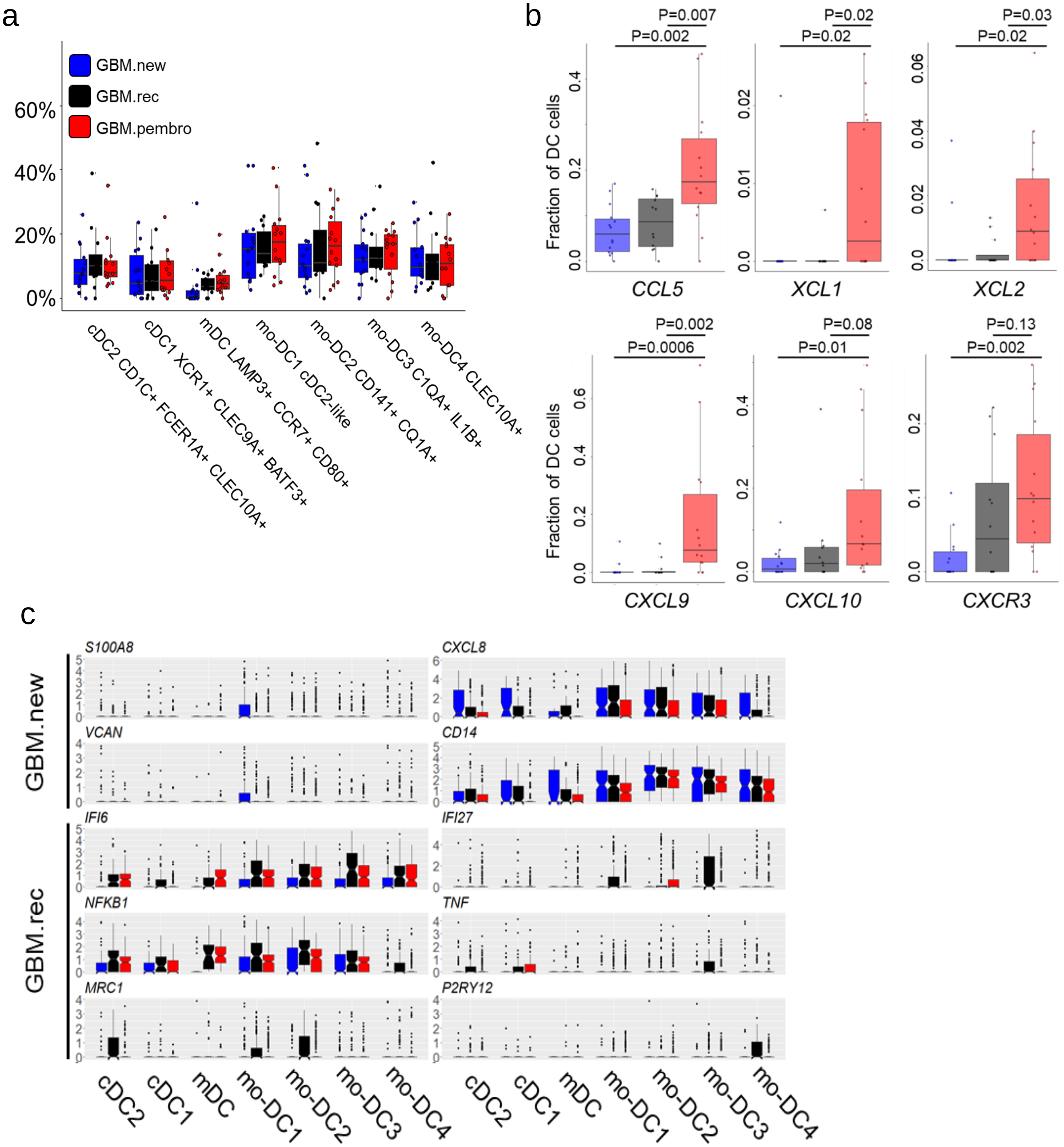

Supplementary Figure 6 – scRNAseq analysis of the dendritic cell populations.

a) The proportion of dendritic cells in each cluster across multiple tumor types (blue: GBM.new, black: GBM.rec, red: GBM.pembro; n=2,960 cells from 40 total patients: 14 GBM.new, 12 GBM.rec, and 14 GBM.pembro).

b) The fraction of dendritic cells with a transcript of indicated genes. Colors are the same as (a).

c) Differentially upregulated genes in GBM.new (top) or GBM.rec (bottom) samples in at least one of the dendritic cell clusters.

P values were calculated using a two-sided Wilcoxon rank sum test. Source data are provided as a Source Data file. In all boxplots, the median is indicated by the line within the box and the 25th and 75th percentiles indicated by the lower and upper bounds of the box. The upper and lower lines above and below the boxes represent the whiskers.

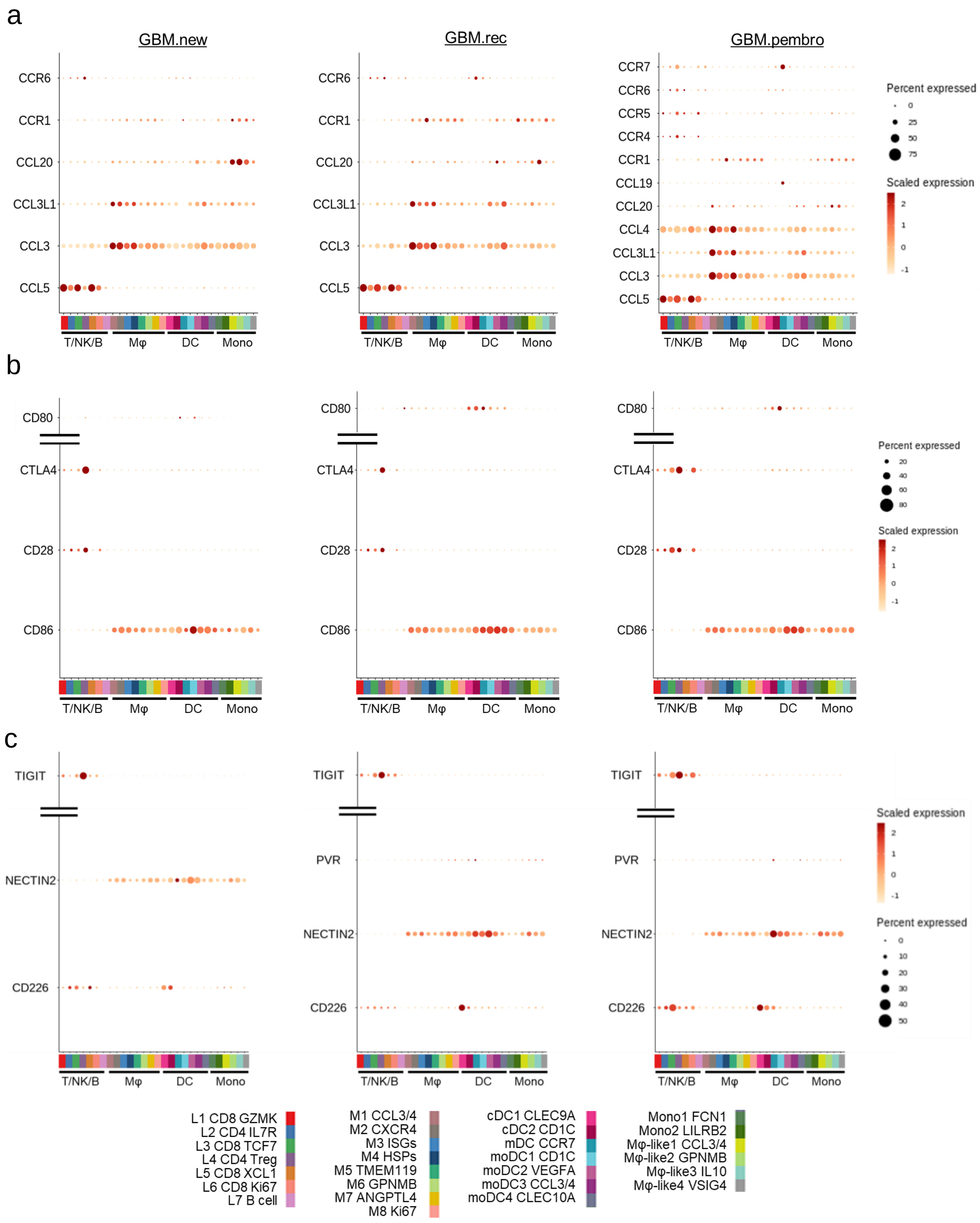

Supplementary Figure 7 – Significantly enriched receptor ligand interactions after neoadjuvant anti-PD1

a) The normalized expression of CCL pathway genes with significant expression in indicated tumor groups.

b) Same as (a) for CD28-CTLA4-CD86 pathway. The expression of CD80 is included from a separate graph including CD28, CTLA4, and CD80.

c) Same as (a) for CD226-PVR-NECTIN2 pathway. The expression of TIGIT is included from a separate graph including TIGIT, PVR, and NECTIN2.

Interactome analysis on the scRNAseq data was conducted using n=156,766 cells from 40 total patients: 14 GBM.new, 12 GBM.rec, and 14 GBM.pembro.

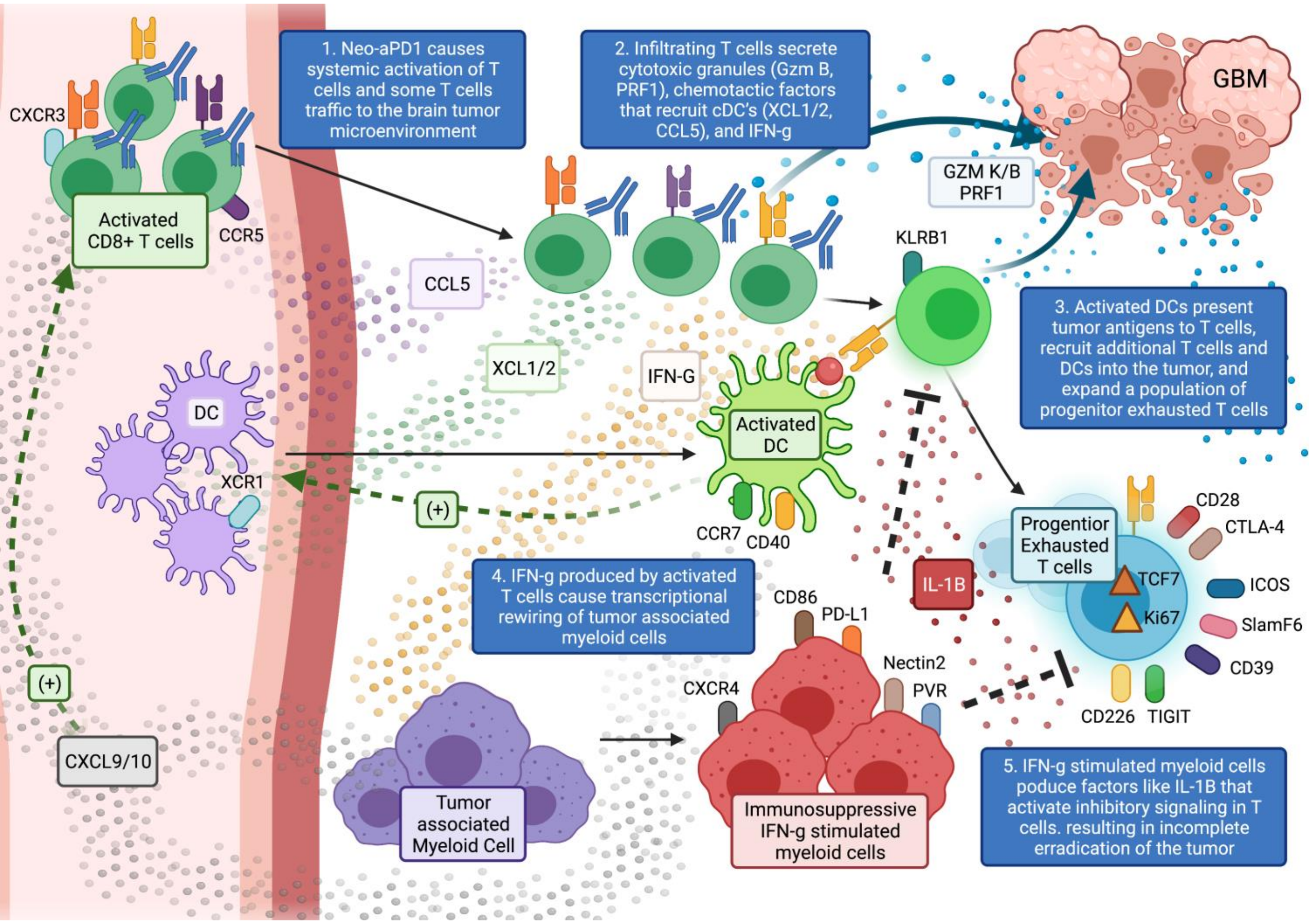

Supplementary Figure 8 - A schematic proposing how neo-aPD1 changes the immune landscape in the recurrent GBM tumor microenvironment. Biorender.com was used to create this schematic.
