# Supplementary material for: Neoadjuvant PD-1 blockade induces T cell and cDC1 activation but fails to overcome the immunosuppressive tumor associated macrophages in recurrent glioblastoma
Source: Nat Commun. 2021 Nov 26;12:6938. doi: 10.1038/s41467-021-26940-2 (PMC8626557; doi:10.1038/s41467-021-26940-2)
Supplement: Supplementary file 3 — Description of Additional Supplementary Files [file 41467_2021_26940_MOESM3_ESM.pdf]

**Title:** Supplementary Data 1
**Description:** Tables of metal conjugated CyTOF antibodies.

**Title:** Supplementary Data 2
**Description:** Seurat based clustering of all intratumoral CD45+ cells analyzed by scRNA-seq (A) the list of gene markers of the cell clusters in Figure 1e, (B) the proportion of cells from each cluster per tumor sample, (C) the difference of each cluster's proportion among GBM.new, GBM.rec and GBM.pembro tumor groups, (D) differentially expressed genes in specified tumor group stratified by the clusters defined in Figure 1e. P values were calculated using a two-sided Wilcoxon rank sum test. FDR based P value adjustment were done in (A) and (D).

**Title:** Supplementary Data 3
**Description:** Seurat based clustering of all intratumoral lymphoid cells analyzed by scRNA-seq (A) the list of gene markers of the lymphoid clusters in Figure 2a, (B) the proportion of cells from each cluster per tumor sample, (C) the difference of each cluster's proportion among GBM.new, GBM.rec and GBM.pembro tumor groups, (D) differentially expressed genes in specified tumor group stratified by the clusters defined in Figure 2a. P values were calculated using a two-sided Wilcoxon rank sum test. FDR based P value adjustment were done in (A) and (D).

**Title:** Supplementary Data 4
**Description:** Seurat based clustering of all peripheral lymphoid cells analyzed by scRNA-seq (A) the list of gene markers of the lymphoid clusters in Figure 3a, (B) differentially expressed genes in specified tumor group stratified by the clusters defined in Figure 3a. P values were calculated using a two-sided Wilcoxon rank sum test. FDR based P value adjustment were done in (A) and (B).

**Title:** Supplementary Data 5
**Description:** The TCRb-based T cell clonotypes in the TIL and peripheral blood lymphoid cells derived using TRUST4.

**Title:** Supplementary Data 6
**Description:** Seurat based clustering of all intratumoral myeloid cells analyzed by scRNA-seq (A) the list of gene markers of the myeloid clusters in Figure 4a, (B) the proportion of cells from each cluster per tumor sample, (C) the difference of each cluster's proportion among GBM.new, GBM.rec and GBM.pembro tumor groups, (D) differentially expressed genes in specified tumor group stratified by the clusters defined in Figure 4a. P values were calculated using a two-sided Wilcoxon rank sum test. FDR based P value adjustment were done in (A) and (D).

**Title:** Supplementary Data 7
**Description:** Differential CyTOF markers expression in the myeloid cell clusters in Supplementary Figure 4a

**Title:** Supplementary Data 8
**Description:** Seurat based clustering of the monocyte subset of the myeloid compartment in the scRNA-seq analysis (A) the list of gene markers of the monocyte clusters in Supplementary Figure 5b, (B) the proportion of cells from each cluster per tumor sample, (C) the difference of each cluster's proportion among GBM.new, GBM.rec and GBM.pembro tumor groups, (D) differentially expressed genes in specified tumor group stratified by the clusters defined in Supplementary Figure 5b. P values were calculated using a two-sided Wilcoxon rank sum test. FDR based P value adjustment were done in (A) and (D).

**Title:** Supplementary Data 9

**Description:** Seurat based clustering of the DC subset of the myeloid compartment in the scRNA-seq analysis (A) the list of gene markers of the DC clusters in Figure 5a, (B) the proportion of cells from each cluster per tumor sample, (C) the difference of each cluster's proportion among GBM.new, GBM.rec and GBM.pembro tumor groups, (D) differentially expressed genes in specified tumor group stratified by the clusters defined in Figure 5a. P values were calculated using a two-sided Wilcoxon rank sum test. FDR based P value adjustment were done in (A) and (D).
