# Supplementary material for: Neoadjuvant PD-1 blockade induces T cell and cDC1 activation but fails to overcome the immunosuppressive tumor associated macrophages in recurrent glioblastoma
Source: Nat Commun. 2021 Nov 26;12:6938. doi: 10.1038/s41467-021-26940-2 (PMC8626557; doi:10.1038/s41467-021-26940-2)
Supplement: Supplementary file 13 — Reporting Summary [file 41467_2021_26940_MOESM13_ESM.pdf]

nature research

Corresponding author(s): Robert Prins, Willy Hugo

Last updated by author(s): Oct 7, 2021

Reporting Summary

Nature Research wishes to improve the reproducibility of the work that we publish. This form provides structure for consistency and transparency in reporting. For further information on Nature Research policies, see our Editorial Policies and the Editorial Policy Checklist.

Statistics

For all statistical analyses, confirm that the following items are present in the figure legend, table legend, main text, or Methods section.

n/a	Confirmed	
[ ]	[x]	The exact sample size (*n*) for each experimental group/condition, given as a discrete number and unit of measurement
[ ]	[x]	A statement on whether measurements were taken from distinct samples or whether the same sample was measured repeatedly
[ ]	[x]	The statistical test(s) used AND whether they are one- or two-sided<br>*Only common tests should be described solely by name; describe more complex techniques in the Methods section.*
[ ]	[x]	A description of all covariates tested
[ ]	[x]	A description of any assumptions or corrections, such as tests of normality and adjustment for multiple comparisons
[x]	[ ]	A full description of the statistical parameters including central tendency (e.g. means) or other basic estimates (e.g. regression coefficient) AND variation (e.g. standard deviation) or associated estimates of uncertainty (e.g. confidence intervals)
[ ]	[x]	For null hypothesis testing, the test statistic (e.g. *F*, *t*, *r*) with confidence intervals, effect sizes, degrees of freedom and *P* value noted<br>*Give P values as exact values whenever suitable.*
[x]	[ ]	For Bayesian analysis, information on the choice of priors and Markov chain Monte Carlo settings
[x]	[ ]	For hierarchical and complex designs, identification of the appropriate level for tests and full reporting of outcomes
[x]	[ ]	Estimates of effect sizes (e.g. Cohen's *d*, Pearson's *r*), indicating how they were calculated

Our web collection on statistics for biologists contains articles on many of the points above.

Software and code

Policy information about availability of computer code

Data collection	Single cell sequencing data was collected by the UCLA TCGB sequencing core using the Illumina NovaSeq platform, mass cytometry data was collected by UCLA JCCC Flow Cytometry core (Helios platform) and the multiple immmunofluorescence were collected at the UCLA TPCL core (Vectra POLARIS platform).
Data analysis	Mass cytometry: Cytofkit 1.4.10 (including the functions Phenograph and ClusterX), Reticulate 1.18, pheatmap 1.0.12, premessa 0.2.4 on R version 3.6.3<br>Single cell RNAseq: CellRanger 3.1.0, Seurat 3.1.5 (including the functions NormalizeData, FindNeighbors, FindMarker), Monocle 2.12.0 on R version 3.6.3<br>TCR: TRUST4 1.0.2, Startrac 0.1.0 on R version 3.6.3<br>Interactome: CellChat R package version 1.1.1 on R version 3.6.3<br>Multiplex immunofluorescence: HALO image analysis platform version v3.0.311.398<br>Scientific visualization: GraphPad Prism version 8.4.0.

For manuscripts utilizing custom algorithms or software that are central to the research but not yet described in published literature, software must be made available to editors and reviewers. We strongly encourage code deposition in a community repository (e.g. GitHub). See the Nature Research guidelines for submitting code & software for further information.

Data

Policy information about availability of data

All manuscripts must include a data availability statement. This statement should provide the following information, where applicable:

- Accession codes, unique identifiers, or web links for publicly available datasets
- A list of figures that have associated raw data
- A description of any restrictions on data availability

Single-cell RNA sequencing raw data files are available in the Gene Expression Omnibus under accession number GSE154795 and CyTOF raw data files are stored at the Flow Repository under the ID FR-FCM-Z4LX. These files supply the data for figures 1-6 and supplementary figures 1-7. The raw single-cell RNA sequencing was aligned to the Genome Reference Consortium Human Build 38 (GRCh38, GCA_000001405.15) prior to analysis. Source data is also provided with this paper. The remainder of data that support the findings of this study, including multiplex IHC images and data files and processed CyTOF fcs files ready for analysis, are available from the corresponding author upon reasonable request.

Field-specific reporting

Please select the one below that is the best fit for your research. If you are not sure, read the appropriate sections before making your selection.

☒ Life sciences ☐ Behavioural & social sciences ☐ Ecological, evolutionary & environmental sciences

For a reference copy of the document with all sections, see nature.com/documents/nr-reporting-summary-flat.pdf

Life sciences study design

All studies must disclose on these points even when the disclosure is negative.

Sample size	In this study, we included all patients where enough CD45+ immune cells were isolated from their surgical specimens and/or peripheral blood samples to complete the described procedures. For CyTOF mass cytometry, we required at minimum input of 500,000 cells, and for single-cell RNA sequencing, we required an input of at least 10,000 cells. No sample size calculations were made and number of cells to analyze were determined by manufacturers' recommendations.
Data exclusions	For CyTOF mass cytometry analysis, only live, intact single cells were analyzed. For single-cell RNA sequencing, dead cells or live cells with low reads and/or low number of features (<200 features/cell) were filtered out.
Replication	Data collected on individual patient samples could not be replicated due to the limited quantity of available specimens.
Randomization	Randomization was not applicable to this study as this study is a post-hoc analysis of the immune landscape of the glioblastoma microenvironment from patient samples separated by their tumor stage and/or immune checkpoint inhibitor treatment
Blinding	Blinding was not applied to this study as the key question of the project was to determine the transcriptional and phenotypic changes associated with different tumor stages and/or with immune checkpoint inhibitor treatment, which required knowledge of each individual patient.

Reporting for specific materials, systems and methods

We require information from authors about some types of materials, experimental systems and methods used in many studies. Here, indicate whether each material, system or method listed is relevant to your study. If you are not sure if a list item applies to your research, read the appropriate section before selecting a response.

Materials & experimental systems

n/a	Involved in the study
☐	☒ Antibodies
☒	☐ Eukaryotic cell lines
☒	☐ Palaeontology and archaeology
☒	☐ Animals and other organisms
☐	☒ Human research participants
☒	☐ Clinical data
☒	☐ Dual use research of concern

Methods

n/a	Involved in the study
☒	☐ ChIP-seq
☒	☐ Flow cytometry
☒	☐ MRI-based neuroimaging

Antibodies

Antibodies used	34 marker CyTOF panel (all 1:50 dilution):<br>115In Ki67 SolA15 eBiosciences 14-5698-95<br>141Pr CD137 (4-1BB) 4B4-1 Biolegend 309802

142Nd CD19 HIB19 Fluidigm 3142001B
143Nd CD25 (IL-2R) M-A251 Biolegend 356102
144Nd CD15 (SSEA-1) W6D3 Fluidigm 3144019B
145Nd CD4 RPA-T4 Fluidigm 3145001B
146Nd CD8 RPA-T8 Biolegend 301002
147Sm CD11c Bu15 Fluidigm 347008B
148Nd TIGIT MBSA43 Thermo Scientific 16-9500-82
149Sm CD278 (ICOS) DX29 BD 7801
150Nd CD152 (CTLA-4) 14D3 Invitrogen 14-1529-82
151Eu CD14 M5E2 Fluidigm 3151009B
152Sm TCRγδ 11F2 Fluidigm 3152008B
153Eu TIM3 F38-2E2 Fluidigm 3153008B
154Sm CD45RO UCHL1 Biolegend 304202
155Gd CD279 (PD-1) EH12.2H Fluidigm 3155009B
156Gd CD274 (PD-L1) 29E.2A3 Fluidigm 3156026B
158Gd CD33 WM53 Fluidigm 3158001B
159Tb CD197 (CCR7) G043H7 Fluidigm 3159003A
160Gd CD39 A1 Fluidigm 3160004B
162Dy CD69 FN50 Fluidigm 3162001B
163Dy CD56 (NCAM) NCAM16.2 Fluidigm 3163007B
164Dy CD38 HIT2 Biolegend 303502
165Ho CD16 B73.1 Fluidigm 3165007B
167Er CD27 O323 Fluidigm 3167002B
168Er CD206 MMR Fluidigm 3168008B
169Tm CD45RA HI100 Fluidigm 3169008B
170Er CD3 UCHT1 Fluidigm 3170001B
171Yb CD68 Y1/82A Fluidigm 3171011B
172Yb CD196 (CCR6) G034E3 Biolegend 353427
174Yb HLA-DR L243 Fluidigm 3174001B
175Lu CD194 (CCR4) L291H4 Fluidigm 3175035A
176Yb CD127 (IL-7Rα) A0195D5 Fluidigm 3176004B
209Bi CD11b ICRF44 Fluidigm 3209003B

Additional CyTOF antibodies not listed above that were used in other panels (all 1:50 dilution):
141Pr TIGIT MBS43 Fluidigm 16-9500-38
144Nd CD69 FN50 Fluidigm 3144018B
148Nd CD274 /PD-L1 29E.2A3 Fluidigm 3148017B
150Nd CD223 (LAG-3) 11C3C65 Fluidigm 3150016B
159Tb CD11c Bu15 Fluidigm 3159001B
161Dy CD152 (CTLA-4) 14D3 Fluidigm 3161004B
164Dy CD15/SSEA-1 W6D3 Fluidigm 3164001B
172Yb CD38 HIT2 Fluidigm 3172007B

mIHC Antibodies
CD14 EPR3653 Abcam ab133335 1:3500
CD206 CL0387 Sigma-Aldrich AMAB90746-25UL 1:300
HLA-DR EPR3692 Abcam ab92511 1:300
CD4 4B12 Dako M731029-2 1:50
CD45 2B11+PD7/26 Dako IR75161-2 1:200
CD8 C8/144B Dako IR62361-2 1:200

Validation

For metal conjugated antibodies used for CyTOF, validation was performed as described by the manufacturer (Fluidigm) or by the UCLA Jonsson Comprehensive Cancer Center (JCCC) Flow Cytometry Core Facility using the appropriate positive and negative cell staining and/or activation controls.
For antibodies used in mIHC analysis, validation was performed by the manufacturers (Abcam, Sigma-Aldrich, Dako) per their listed validation protocols.

Human research participants

Policy information about studies involving human research participants

Population characteristics	Patients were aged ≥ 18 years, male and female, of all ethnicities, having World Health Organization grade II-IV malignant glioma that were candidates for surgical debulking. Key eligibility criteria included Karnofsky performance status ≥70, adequate organ function, and absence of previous anti-angiogenic or anti-vascular endothelial growth factor agents.
Recruitment	Participants were not specifically recruited for the purpose of this study; samples were included based on tumor diagnosis (GBM), the availability of the specimen, and whether sufficient amount of live cells were collected from the specimen to allow for accurate single cell analyses. We analyzed all samples that fulfilled the above criteria so there were no/minimal self-selection bias.
Ethics oversight	Written informed consent was provided by each patient. The UCLA Medical Institutional Review Board 2 (IRB#10-000655-AM-00059) approved all protocols related to patient specimen collection.

Note that full information on the approval of the study protocol must also be provided in the manuscript.
